# Safety and Tolerability of Topical Ophthalmic Triamcinolone Acetonide-Loaded Liposomes Formulation and Evaluation of Its Biologic Activity in Patients with Diabetic Macular Edema

**DOI:** 10.3390/pharmaceutics13030322

**Published:** 2021-03-02

**Authors:** Jose Navarro-Partida, Juan Carlos Altamirano-Vallejo, Alejandro Gonzalez-De la Rosa, Juan Armendariz-Borunda, Carlos Rodrigo Castro-Castaneda, Arturo Santos

**Affiliations:** 1 Tecnologico de Monterrey, Escuela de Medicina y Ciencias de la Salud, Campus Guadalajara, P.C. 45138 Zapopan, Mexico; josenavarro@tec.mx (J.N.-P.); jcaltamirano@e-retina.com (J.C.A.-V.); agonzalez1@tec.mx (A.G.-D.l.R.); armendbo@cucs.udg.mx (J.A.-B.); crodrigocastro@gmail.com (C.R.C.-C.); 2 Centro de Retina Medica y Quirurgica, S.C., Centro Medico Puerta de Hierro, P.C. 45116 Zapopan, Mexico; 3 Instituto de Biología Molecular y Terapia Genica, Centro Universitario de Ciencias de la Salud, Universidad de Guadalajara, P.C. 44340 Guadalajara, Mexico

**Keywords:** ocular drug delivery system, topical liposomes, posterior segment of the eye, safety and tolerability, biologic activity, diabetic macular edema

## Abstract

Intravitreal injections (IVTs) of corticosteroids as triamcinolone acetonide (TA) are frequently used for the treatment of many vitreous and retinal disorders. However, IVTs are related to severe ocular complications. Lately, a topical ophthalmic TA-loaded liposomes formulation (TALF) was designed to transport TA into the posterior segment of the eye when instilled on the ocular surface. To evaluate the safety, tolerability, and biological activity of TALF, an animal study and a phase I clinical assay were performed. Moreover, four patients with diabetic macular edema (DME) were treated with TALF in order to explore the biological activity of the formulation. No inflammation, lens opacity, swelling, or increase in intraocular pressure were recorded after the instillation of TALF in any of the animal or clinical studies. Mainly, mild and transient adverse events such as dry eye and burning were reported. TALF significantly improves visual acuity and diminishes central foveal thickness in patients with DME. The current data demonstrate the safety, tolerability, and biological activity of TALF. It seems that TALF can be used topically to treat vitreous and retinal diseases that respond to TA such as DME, avoiding the use of corticosteroid IVTs and their associated hazards.

## 1. Introduction

Intravitreal injections of corticosteroids are commonly used for the treatment of many vitreous and retinal disorders such as retinal vein occlusions [1], uveitis [2], and diabetic macular edema (DME) [3]. Among the different synthetic corticosteroids, triamcinolone acetonide (TA) is extensively used in intravitreal injections (IVTs). In addition its advantages such as its low cost, TA is not exempt from severe complications, including a detached retina, endophthalmitis, and vitreous hemorrhage [4,5,6,7], as well as the risk of severe and intractable intraocular pressure (IOP) elevation [8,9,10], which raises constant concerns about intraocular TA administration. In addition, the discomfort caused to the patient by IVT itself could cause poor compliance with intraocular steroid treatment [11,12,13]. 

Although oral or topical corticosteroid routes of administration could be safer than IVTs, these have limited effectiveness for vitreoretinal disorders. Due to the blood–retinal barriers, these routes scarcely reach the posterior segment of the eye [14]. Therefore, to diminish the ocular risks associated with intravitreal injections, different topical approaches have been developed to deliver TA into the vitreous cavity [15,16,17,18]. Among these, topical liposomes (LPs) are one of the most promising strategies [19]. In fact, LPs constitute the only topical system to deliver TA into the vitreous area with clinical evidence of its effectiveness [20,21,22]. The vesicle composition of LPs share almost an indistinguishable outer structure with the cell membrane, conferring its ability to transport either hydrophilic or lipophilic drugs. LPs represent a potential alternative for ocular drug delivery based on their various advantages—increased residence time for drug absorption, protection of the encapsulated drug from the external environment, prolonged half-lives in vitreous bodies with low toxicity, increased efficacy and therapeutic drug index, and the potential to improve penetration to ocular tissues [19].

In recent years, different topical liposomes have been developed to deliver drugs into the posterior segment of the eye [23,24,25,26,27,28,29,30,31]. For example, fluorescent molecules like 5(6)-carboxyfluorescein and coumarin-6 [23,25,29,30], drugs such as edaravone and diclofenac [26,27], and biomolecules such as nucleic acids [28,31] and bevacizumab (a monoclonal antibody against vascular endothelial growth factor A) [24] have been released into the vitreous body and retina by liposomes.

Recently, a topical ophthalmic triamcinolone acetonide-loaded liposome formulation (TALF) was designed for our group to transport TA into the posterior segment of the eye. The efficiency of this drug delivery nanosystem was successfully tested using in vitro and in vivo models [18]. The in vitro diffusion assay of TALF was performed using diffusion chambers with rabbit corneas as membranes, whereas the in vivo assay was carried out in New Zealand white rabbits. As exposed in the previous report [18], TALF was able to cross the cornea and to deliver TA to the vitreous body and retina, reaching the highest peak at 12 h (32.6 ± 10.2 ng/g and 252.1 ± 90.0 ng/g respectively). Additionally, in the same report, TALF underwent a physicochemical characterization, as well as a cell toxicity assay using primary human corneal fibroblast cultures. We found that TALF has a pH of 5.8, viscosity of 70 cP and osmolarity of 334 mOsm/L. Therefore, TALF was found to be highly viscous, near to the physiologic pH of tears (6.5 to 7.6) [32] and non-irritating to the eye according to the criteria of the *Pharmacopoeia of Estados Unidos Mexicanos*, in which a formulation is considered suitable for ophthalmic use when its osmolarity is between 205 and 684 mOsmol/L. Moreover, cell viability was unaffected by TALF. Finally, the microscopic characterization of TALF using scanning electron microscopy (SEM) and transmission electron microscopy (TEM) revealed that TALF is capable of solubilizing large TA crystals into nanoparticles and encapsulating them at the same time [22].

After an extensive physicochemical, microscopic, and pharmacokinetic characterization of TALF by our group, the purpose of the present study was to effectively evaluate its ocular safety and tolerability in animals, as well as its safety and biological activity in patients with diabetic macular edema, in order to establish the feasibility of the potential clinical use of TALF.

## 2. Materials and Methods

### 2.1. Preparation of TALF 

A self-forming and thermodynamically stable liposome platform (QuSomes^®^, OPKO Health Inc., Miami, FL, USA) was used as a carrier for TA. OPKO Health Inc., Guadalajara, Mexico, provided the TALF. The preparation of TALF was performed in good manufacturing practice (GMP) facilities and was carried out as previously described [18]. Briefly, triamcinolone acetonide is first added to a lipid mixture containing polyethyleneglycol (PEG-12) glyceryl dimyristate and ethyl alcohol. An aqueous mixture containing grade 2 purified water, polyethylene glycol (15)-hydroxystearate (Kolliphor HS 15), citric acid anhydrous, sodium citrate dehydrate, and benzalkonium chloride is commingled in a flask and set aside for compounding. The water mixture is gently added to the lipid mixture to obtain the final formulation. Nitrogen pressure (≤200 psi) was implemented for the extraction of TALF for a 10-cycle duration using a 0.22-µm pore size polycarbonate membrane in order to ensure the least size variability between lipid vesicles. A pH of 5.8 was achieved in the final formulation, with a 70 cP viscosity and an osmolarity of 334 mOsm/L. The size of the loaded liposomes was 187.8 nm (Zetasizer Nano ZS; Malvern Instruments, Malvern, UK). The composition of TALF is provided in Table 1. The final TA concentration was 2 mg/mL (0.2%) in the resultant aqueous suspension [18].

### 2.2. Evaluation of Safety and Tolerability of TALF in Animals

Thirty-two male New Zealand white rabbits, weighing 2–2.5 kg each and free of any sign of ocular inflammation or gross abnormality, were used. The animals were housed individually in standard cages with controlled conditions, exposed to a 12 h dark/12 h light cycle, with continuously ventilated rooms at constant, identical, and defined room temperature (18 ± 3 °C) and humidity (45–75% relative humidity). The rabbits received a standard dry pellet diet and water ad libitum.

Animals received 1 drop (50 μL) of TALF every two hours 6 times daily in the right eyes, also called study eyes (only in the 12-h of light period), and an eye clinical evaluation was carried out under anesthesia at 10, 30, and 60 min; 6, 12, and 24 h; and 7 and 14 days after treatment. Anesthesia was achieved using an intramuscular injection of ketamine hydrochloride 30 mg/kg and chlorpromazine hydrochloride 15 mg/kg. The control eyes (left eyes) received one drop (50 μL) of placebo solution (saline balanced solution) in the same frequency as TALF was applied. TALF and placebo solution were stored at controlled room temperature (18 °C ± 3 °C) in the interim. Four rabbits were euthanized posterior to each clinical evaluation to obtain ocular tissues and fluids, which were placed under freezing conditions at −70 °C, until their storage and subsequent shipment to the Research and Pharmaceutical Research and Development Laboratory facilities (PRDL) to determinate the TA concentration in them. TA levels were analyzed by means of high-performance liquid chromatography (HPLC) at 30 °C. The pharmacokinetic findings were described in a previous report [18]. 

The potential ocular irritancy and/or damaging effects of the formulation (TALF) were evaluated at each established time according to a modified Draize test [33]. The Draize test is an evaluation for the harmfulness of chemicals to the human eye that invalves dropping the test substance into one eye of a rabbit without anesthesia, using the other eye as a control. A slit lamp (CSO Elite, Stagnacci, Firenze, Italy) was used for the ophthalmic evaluation of rabbits. Congestion, swelling, and discharge of the conjunctiva were graded on scales from 0 to 3, 0 to 4, and 0 to 3, respectively (0 was considered normal). Iris hyperemia and lens opacity were also graded on a scale from 0 to 4 (0 was considered normal). Ophthalmic evaluation of the rabbits also included fluorescein (AK-Fluor^®^ Akorn, Lake Forest, IL, USA) and lissamine green (Rose Stone Enterprises, Alta Loma, CA, US) stains, for evaluation of the cornea and conjunctiva, respectively. The ocular surface stains were performed under topical anesthesia with tetracaine hydroclholide 0.5% (Ponti^®^, Laboratorios SOPHIA, Jal, Mexico). For lissamine green (LG) staining, 1.5-mg impregnated strips of 1% of the reagent were used. These were moistened with about 10–20 uL of saline and applied to the lower fornix. The time for evaluating conjunctival staining was between two and four minutes after the instillation to avoid instant viewing of the staining pattern, which could result in misinterpretation due to any pooled dye which had not dissipated. The number of conjunctival spots (if any) stained with LG were reported. For fluorescein staining of the cornea, fluorescein solution at 2% was applied six minutes following the instillation (and clinical examination) of LG. The number of corneal spots (if any) stained with fluorescein was reported.

Based on the *Pharmacopoeia of Estados Unidos Mexicanos* document, the presence of ocular irritation was evaluated. The existence of characteristic findings of irritation, such as inflammation of the iris or conjunctiva, conjunctival vessel dilation, corneal ulceration, and/or opacity in more than one animal, was considered a positive test result.

Furthermore, intraocular pressure measurement (IOP) (Icare^®^ TA01i tonometer, Belleville, MI, USA) and a fundus evaluation with binocular indirect ophthalmoscope (Killer Vantage Plus LED, Malvern, PA, USA) were performed.

### 2.3. Evaluation of Safety and Tolerability in Healthy Volunteers

A phase I clinical assay was designed to evaluate the safety and local tolerability of TALF upon repeated-dose topical application to one eye in healthy male and female volunteers between 18 and 60 years old. Healthy subjects were defined as having the absence of medical and surgical history (except cataract surgery) in their medical records. Healthy eyes were defined as best corrected visual acuity (BCVA) >80 letters in the Early Treatment Diabetic Retinopathy Study (ETDRS) chart, tear film rupture time >9 s, unanesthetized Schirmer test ≥10 mm, and central foveal thickness (CFT) <300 μm (measured using optical coherence tomography (OCT; Cirrus OCT Carl Zeiss, Meditech, Dublin, CA, USA)). 

Abnormal slit lamp ophthalmologic examination result, defined as the presence of dry eye syndrome, previous trauma, eyelid pathologies (blepharitis, glandular dysfunctions) or Stevens–Johnson syndrome, were part of the exclusion criteria. Subjects were also excluded if they had a history of any intraocular surgery (except cataract surgery at least 3 months before baseline visit) or ocular laser surgery. Subjects were not included if current allergic reactions, active infection, either ocular or systemic, or a 3-month previous screening history of contact lens usage were present. Eye suitability was dependent on the fulfilment of the inclusion criteria and the rejection of the exclusion criteria. Finally, subjects were also excluded if an abnormal tear break-up time or Schirmer test on each eye, as well as a dry eye syndrome defined by the ocular surface disease index (OSDI), was present at the screening.

Demographic and baseline clinical exams were collected for every volunteer 1–5 days before the use of TALF. The study eye was randomly selected. Subjects who met all eligibility requirements began the study with a TALF instillation regimen. All the healthy volunteers were instructed to apply one drop of TALF 6 times a day for 2 weeks. Follow up was extended for another week to monitor local and systemic tolerability (week 3), for a total of 21 days of follow up. Compliance of the patient to TALF instillation was analyzed through a patient care journal as follows:

$$\mathrm{AD}\;=\;\frac{\mathrm{RA}\left(100\right)}{\mathrm{IA}}$$

where AD is adherence, RA corresponds to the registered applications, and IA represents the indicated number of applications. Compliance failure was considered with a value of adherence lower than 80%. In the case of compliance failure, the patient was excluded from the statistical analysis.

COFEPRIS guidelines were used for general safety assessments in vital sign monitoring. Information gathered from patients, personnel, or elsewhere about the ocular, non-ocular, or serious adverse events (SAEs), as well as vital signs and ocular examinations, determined the standards regarding tolerability. If evident adverse events (AEs), especially involving the anterior segment or poor tolerability, were recorded in relation to the application of TALF, subjects were removed from the study. Verification of drug severity and its relationship was verified by the examiner. The MedDRA coding dictionary, version 18.1, was used for the assignment of AE standard codes. In case any AEs were detected, the subject was instructed to stop the research drug and was excluded from the study.

The safety and ocular tolerability assessments of the *Farmacopea de los Estados Unidos Mexicanos 2018* (FEUM) were also included. Ocular AEs were reported in obedience to NOM-220-SSA1-2016, which establishes the Mexican regulatory guidelines for instillation and handling of research and commercial drugs and its adverse events. In accordance with this guideline, ocular AEs must be reported using establishing degrees—mild (no treatment is needed), moderate (specific treatment may be needed, study drug is not suspended), and severe (specific treatment is needed, study drug is suspended).

Other ocular examinations included BCVA, assessed at 4 m using standardized procedures based on the ETDRS protocol; contrast sensitivity (CS), evaluated using the Pelli–Robson contrast sensitivity test; intraocular pressure (IOP), measured using a Goldmann Applanation Tonometer; corneal endothelial cell density (cECD), determined by specular microscopy (Perseus endothelial microscope, Costruzione Strumenti Oftalmici, Firenze, Italy); and retinal thickness and structural changes of the retina, evaluated by means of OCT before and after treatment.

### 2.4. Evaluation of the Biologic Activity of TALF in Patients with Diabetic Macular Edema

A single center, single arm, open-label, prospective, nonrandomized study was performed to evaluate biological activity in patients with DME. The inclusion criteria were as follows—glycated hemoglobin (HbA1c) less than 6.5%, diagnosis of DME as defined by ETDRS [34], BCVA equal or worse than 20/40 Snellen equivalent, and CFT equal to or more than 250 μm measured by OCT (Cirrus OCT Carl Zeiss, Meditech, Dublin, CA, USA). The exclusion criteria included proliferative diabetic retinopathy, ocular hypertension or glaucoma, previous vitreo-retinal surgery, laser photocoagulation, and/or IVT in the study eye within 3 months prior to enrollment.

Two weeks prior to initiating the intervention, data from selected participants regarding demographics and initial clinical exams were gathered. Instructions regarding TALF application and preservation of drops were delivered to the patients. The indication was to apply the formulation 6 times a day and to keep the dropper bottle containing TALF at room temperature. TALF therapy was applied for 6 months. Follow-up visits were scheduled monthly. An ophthalmic clinical evaluation was performed during each visit. This evaluation included BCVA estimation using the ETDRS chart at 4 m, IOP measurement, anterior segment and retina observation with a slit-lamp, and CFT measurement by OCT. In addition, the AEs were recorded as previously stated. It is important to emphasize that a new dropper containing TALF was provided during each follow-up visit. 

Rescue therapy with 0.5 mg of intravitreal ranibizumab (Lucentis^®^, Novartis Farmacéutica, S.A. de C.V., Ciudad de Mexico, Mexico) was considered if a diminution in visual acuity was registered (five letters or more in the ETDRS chart) compared to a previous visit, or if an increase was recorded in the CFT of 50 μm or more, measured by OCT, compared to a previous visit.

### 2.5. Ethical Considerations

The animal assay met the guidelines of the Association for Research in Vision and Ophthalmology (ARVO) and the guidelines from the 2010/63/UE European Convention for the Protection of Vertebrate Animals used for Experimental and Other Scientific Purposes. The clinical studies were conducted at a private-based retina specialty center in Guadalajara, Mexico (Centro de Retina Medica y Quirurgica S.C.). Institutional Review Board (IRB)/Ethics Committee approval was obtained before the enrollment of patients (IJICSA Committee; ID: CRMQ-2015-05-T-02; approved on 13 December 2016; COFEPRIS 173300410A0035/2017). The studies were implemented in accordance with the tenets of the Declaration of Helsinki. Written informed consent was obtained from all patients.

### 2.6. Statistical Analysis

Quantitative variables are presented as means ± standard deviations of the mean. Qualitative variables are described using frequencies and percentages. For the analysis of differences, a Fisher exact test or a Friedman test of repeated measures was performed. Significance was defined as a *p*-value less than 0.05. To analyze the data, we used the SPSS 22.0 software (IBM SPSS Statistics for Macintosh, Version 22.0 (IBM Corp, Armonk, NY, USA). 

## 3. Results

### 3.1. TALF Is Well Tolerated in the Preclinical Model

**Figure 1:**
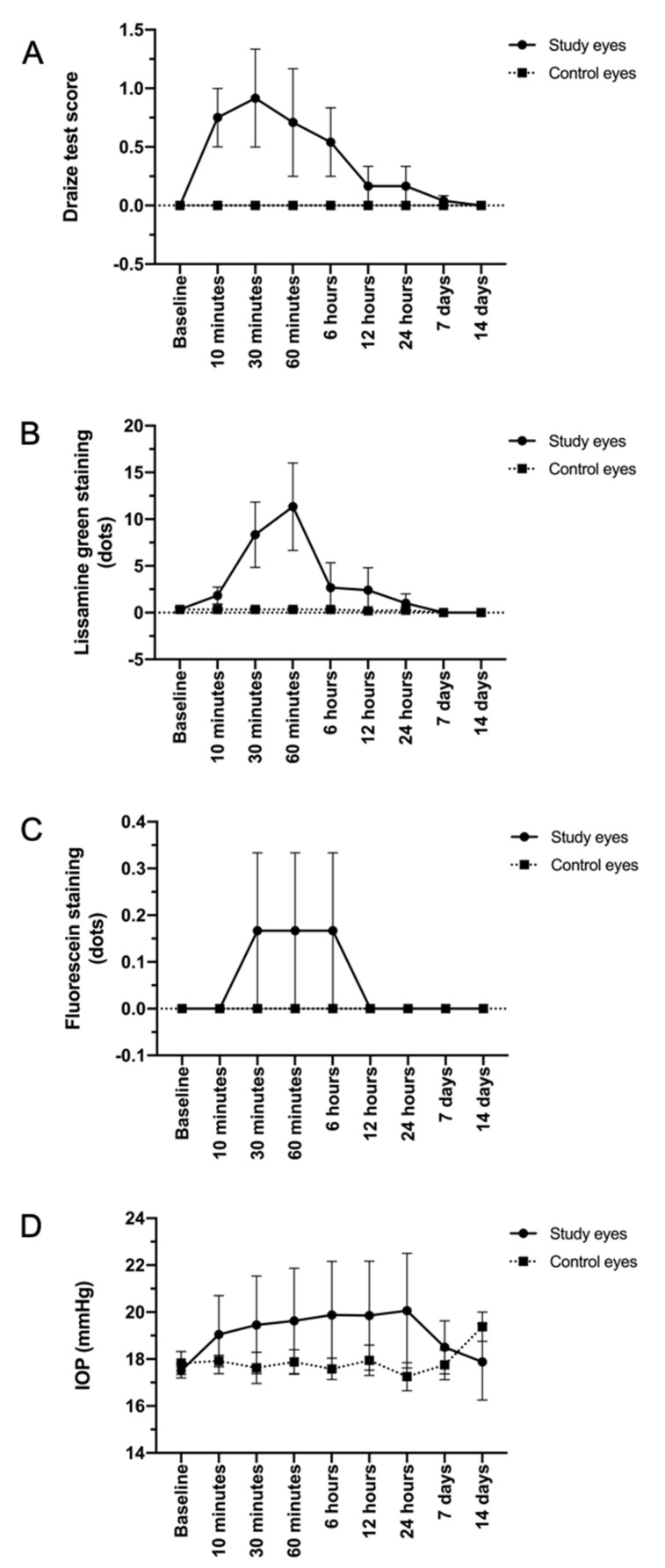
Mean Draize test scores (**A**), staining scores (**B**,**C**), and intraocular pressure (**D**) in study and control eyes. Non-significant differences were observed between eyes treated with triamcinolone acetonide-loaded liposomes formulation and control eyes receiving an instillation of saline-balanced solution during the follow-up.

Examination of the ocular surface (cornea and conjunctiva), as well as the inner parts of the eye (iris, lens, and retina) of rabbits was performed in order to characterize the potential side effects that may result from a high number of daily instillations of TALF. Ocular examination did not show any major findings or adverse events through the follow-up. Figure 1 shows the mean Draize test score for the study and control eyes at different points of the follow-up.

**Figure 2:**
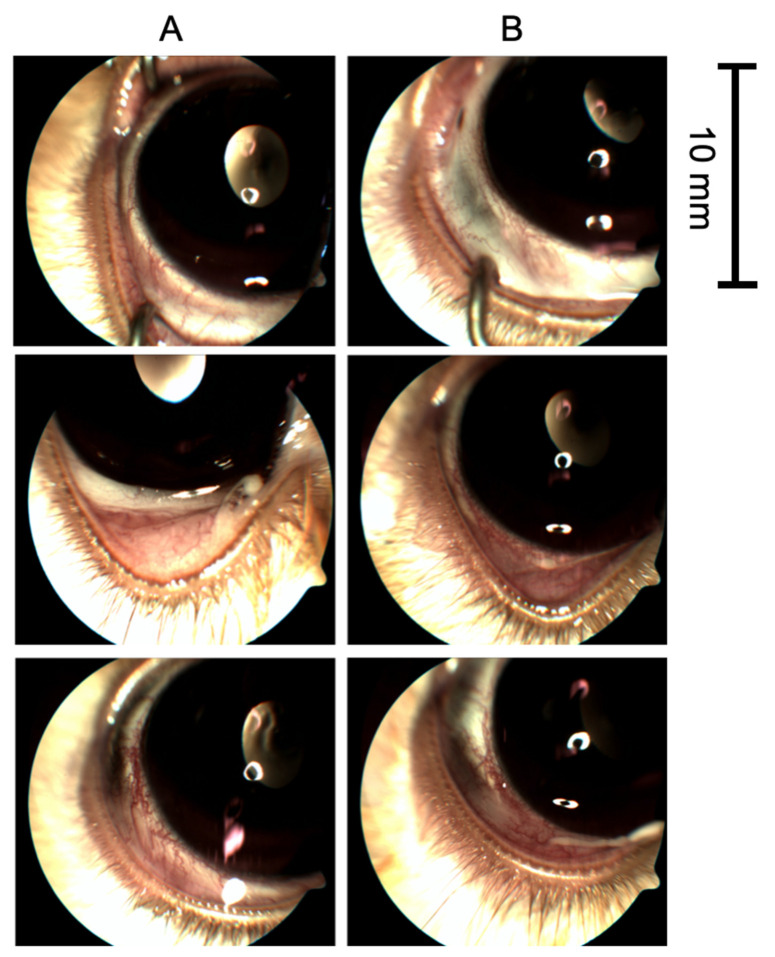
Representative images of study and control eyes. (**A**) Study eyes and (**B**) control eyes photographs show no inflammation, swelling, or discharge after the instillation of triamcinolone acetonide-loaded liposomes formulation (TALF) or placebo solution, respectively.

To quantify the ocular irritation potential of TALF, the parameters of congestion, swelling, and discharge of conjunctiva, iris, hyperemia, and corneal opacity were evaluated in the rabbits. Since the first instillation of TALF and until the end of the study, there was no evidence of inflammation, tissue alteration, and/or discomfort in rabbit eyes. A zero score (0) was achieved in all eyes for inflammation. For swelling and conjunctival congestion, a zero score was also registered at the end of the follow-up in all groups. Iris hyperemia and corneal opacity scores were also equal to zero at all observations. We did not observe changes in the lens during the follow-up of the animal groups. Representative images of study and control eyes that show no inflammation, swelling, or discharge after the instillation of TALF or placebo are presented in Figure 2.

Staining with fluorescein and lissamine green showed superficial epithelium punctate keratitis in the first 6 h after instillation of the formulation. This condition was resolved in all cases in the examination at 12 h after the administration of the formulation. Staining scores of ocular surfaces are presented in Figure 1.

Finally, no increase in intraocular pressure was observed in any of the study animals (normal intraocular pressure in this species is 12–28 mmHg). The behavior of IOP is presented in Figure 1.

### 3.2. TALF Is Safe for Healthy Volunteers

Twenty healthy volunteers were enrolled in the study. The mean age was 37 ± 14.8 years. Thirteen (65%) subjects were women and seven (35%) were men. Fifteen of the 20 study eyes were right and five were left. All subject demographics and characteristics are summarized in Table 2.

In relation to safety and tolerability outcomes, we observed that the TALF was well tolerated during the study period. No systemic AEs were reported. None of the 20 patients showed significant changes in IOP, BCVA, contrast sensitivity, or CFT (Table 2). After using the study formulation, none of the patients required treatment with IOP-lowering drugs. No serious AEs were reported (Table 3) after the end of study drug application (day 14) and at the end of the study follow-up (day 21).

**Figure 3:**
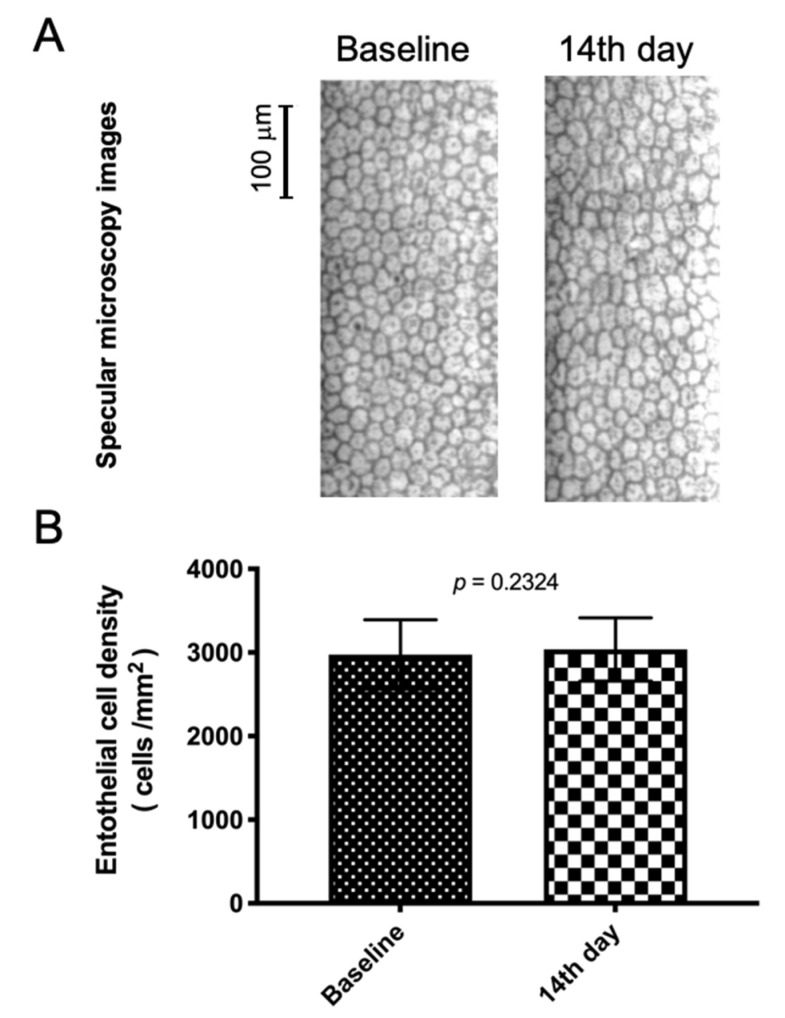
Corneal endothelial cell density analysis in healthy subjects treated with triamcinolone acetonide-loaded liposomes formulation. (**A**) Images of specular microscopy of a representative case at baseline and after 14 days of TALF instillation. (**B**) Column bar graph from corneal endothelial cell density (cECD) analysis. Non-significant differences in cECD values were established between baseline and after 14 days of TALF instillation. TALF; triamcinolone acetonide-loaded liposomes formulation.

Ocular AEs were reported according to the NOM-220-SSA1-2016 as a COFEPRIS request. Most AEs were mild and two of them were moderate in severity. Six subjects (30%) reported occasional mild dryness on one occasion and six others (30%) reported a mild burning sensation (one or two times) during the instillation. Two volunteers (10%) reported moderate secretions that disappeared after the first week of the study and three (15%) reported tearing at the moment of application. A relationship with the drug could not be excluded. There was no pain or discomfort reported. Neither eyelid redness, conjunctival hyperemia, nor edema were observed across the follow-up. No subconjunctival hemorrhages were reported in this study. The examiner acknowledged the nonsignificant results ranked from 0 (no changes) to 1 (mild changes) in relation to the ocular surface staining. The anterior eye chamber and lens showed no abnormal findings. Vitreous cells and flares were absent. Retinal normal appearance remained unchanged before and after intervention. Endothelial cell concentration and retinal condensation were unaltered (2976 ± 414 cells/mm^2^ vs. 3037 ± 377 cells/mm^2^) (Figure 3). No clinically relevant changes in vital sign parameters were observed. TALF eye drops did not affect blood pressure or pulse rate. No local or systemic findings required TALF to be stopped. A summary of reported ocular AEs are presented in Table 3.

### 3.3. TALF Improves BCVA and Reduces CFT in Patients with DME

Four patients with DME were enrolled in the study. The mean age was 62 ± 7.87 years. Two patients (50%) were women. All study eyes were left eyes.

Concerning safety and tolerability outcomes, we observed that the TALF was well tolerated during the study period. No systemic or severe AEs were reported. None of the patients showed significant changes in IOP. After using TALF for 6 months, none of the patients showed intraocular hypertension.

**Figure 4:**
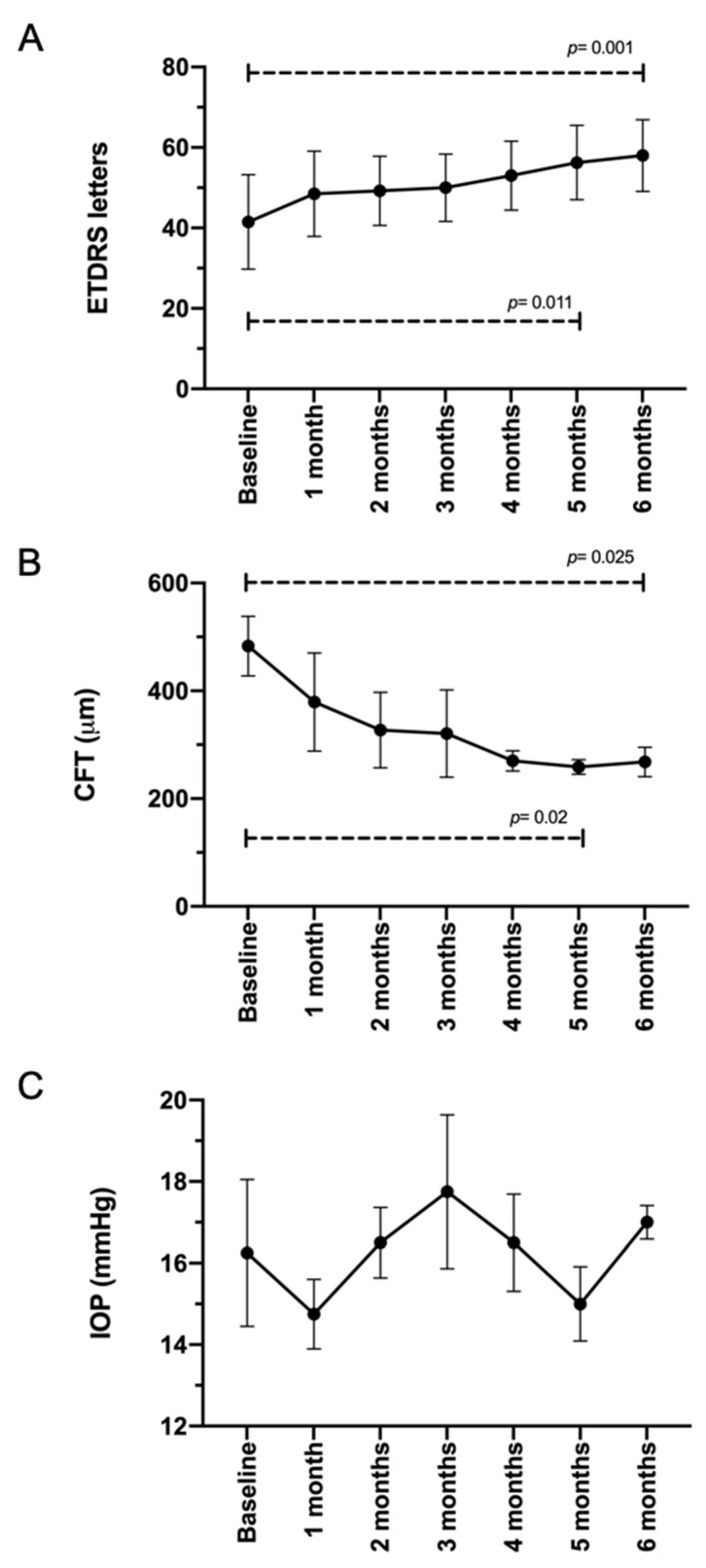
Best corrected visual acuity, central foveal thickness, and intraocular pressure follow-up in patients with diabetic macular edema treated with triamcinolone acetonide-loaded liposomes formulation. (**A**) Best corrected visual acuity, measured by ETDRS test, improved through the follow-up, reaching statistical significance at month 5. (**B**) CFT, measured by OCT, contracted through the follow-up, accomplishing statistical significance at month 4. (**C**) Non-significant variation in IOP was recorded during the study. ETDRS; Early Treatment Diabetic Retinopathy Study, CFT; central foveal thickness, OCT; optical coherence tomography.

**Figure 5:**
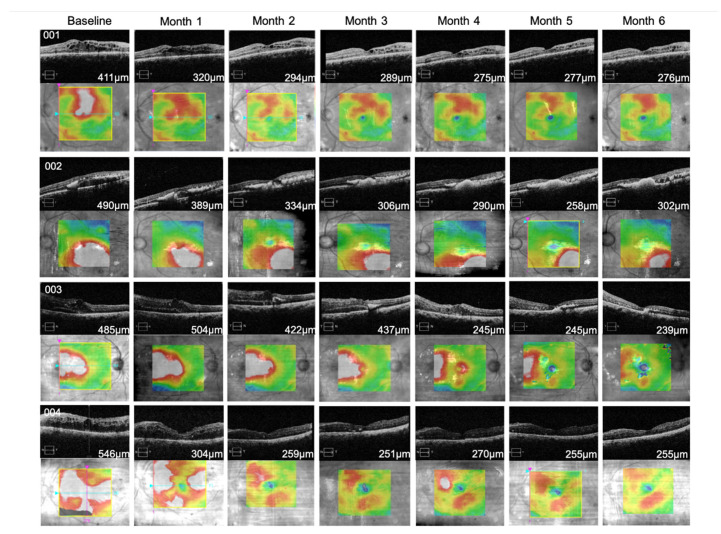
Optical coherence tomography images from patients with diabetic macular edema treated with triamcinolone acetonide-loaded liposomes formulation. A significant reduction in CFT was revealed through OCT analysis in patients receiving TALF. The CFT reduction was maintained through the follow-up in almost all cases. CFT; central foveal thickness, OCT; optical coherence tomography, TALF; triamcinolone acetonide-loaded liposomes formulation.

On the other hand, BCVA and CFT improved significantly over time (Figure 4). ETDRS letter scores increased from 41.5 ± 23.5 at baseline to 58 ± 17.79 letters after 6 months of TALF therapy (*p* = 0.001), whereas CFT measured by OCT decreased from 483 ± 55.39 at baseline to 268 ± 27.26 μm at the same point (*p* = 0.025). OCT images from all cases are provided in Figure 5. Additionally, no patients required rescue therapy. 

## 4. Discussion

Over the last decade of research, dramatic changes have been observed in the field of drug delivery. Developing a novel drug delivery system to target a particular human tissue has become a major goal for researchers in the field [35,36]. Although there has been great interest in the development of new topical ocular delivery systems, ocular drug delivery has been a major challenge to scientists due to its unique anatomy and physiology. Topical ophthalmic drug molecules must cross several layers of eye tissue and trespass several dynamic and metabolic barriers before reaching the vitreous body and retina, and very low concentrations with almost no clinical effect are usually obtained [36,37]. This is why conventional topical administration of drugs has not been as effective as intravitreal delivery in the treatment of retinal diseases [36].

Nowadays, IVTs are the most used pathway to deliver drugs for the posterior segment of the eye and it has become the most common intraocular procedure worldwide, with increasing numbers every year [38]. IVTs are the standard drug delivery method for the treatment of retinal diseases that cause non-reversible vision impairment [37,39]. The advantage of the intravitreal method is to circumvent the ocular barriers which keep most drugs out of the eye, and the ability to minimize the loss of vision and to improve it over a period of years is an untold clinical benefit [40]. Due to the widespread use of IVT, new therapeutic opportunities for previously untreated ophthalmic patients have improved their vision using drugs already known to the market, such as anti-vascular endothelial growth factor (VEGF), VEGF trap-eye, and triamcinolone [13].

However, IVTs are not exempt from potential problems. The need for multiple consecutive injections increases the incidence of adverse events (AEs) such as endophthalmitis, lens injury, and retinal detachment over the months and years of use [11,12,13,41,42]. In addition, it may be a burden for patients, physicians, and health systems, with poor compliance in many cases [11,12,13]. In addition, IVT requires highly specialized human resources and special infrastructure for its application, making it a costly option in developing countries [13]. Scientists, organizations, and pharmaceutical companies worldwide are continuously looking for a safer, more accessible, and effective ocular drug delivery method for ocular use.

Different implantable and non-implantable [43,44,45,46,47,48,49,50,51] delivery systems, as well as novel ocular drug delivery systems such as microemulsions, nanosuspensions, nanoparticles, and liposome formulations [52,53,54], have been analyzed in order to reduce side effects and reach better patient compliance, to bring about better outcomes. In a previous work, we showed that TALF, a novel system used to deliver TA to the posterior segment of the eye, reaches significant concentrations in the posterior segment of the intraocular tissues and is non-toxic for human keratocyte cultures [18], but the in vivo safety and tolerability of this formulation was still a concern, and the biological and therapeutic effects on a chronic retinal disorder such as diabetic macular edema had not been explored previously. Thus, this report summarizes the findings about the biological activity, safety, and tolerability of TALF.

With respect to the biological activity, we found that TALF was able to reduce the thickness of the fovea (measured by OCT) in patients with adequate glycemic control but the presence of DME. This finding is not surprising because the benefits of synthetic corticosteroids for the therapy of DME are well-known due its numerous targets, but the remarkable finding is the administration route. TALF is the first liposome formulation carrying TA that has shown therapeutic activity clinically. Although a complete understanding of the mechanism of action of corticosteroids has not been fully elucidated, it has been demonstrated that corticosteroids, like the TA contained in TALF, interfere with regulatory components of gene expression, inhibiting the synthesis of vascular endothelial growth factor (VEGF) and multiple pro-inflammatory genes such as tumor necrosis factor α and interleukin 6, while inducing anti-inflammatory factors such as pigment epithelium-derived growth factor (PEDF) [55,56,57]. Furthermore, steroids also inhibit the phospholipase A2 pathway, diminishing the production and release of inflammatory cell mediators and chemokines. Moreover, TA specifically seems to reduce the expression of matrix metalloproteinases (MMPs) and downregulates intercellular adhesion molecule 1 in choroidal endothelial cells [58].

On the other hand, ocular tolerability is a major goal for all topical drugs. Many eye conditions are treated with ophthalmic topical formulations with good results, but tolerability is still a concern for many patients. In our animal study, we did not observe superficial irritation signs such as conjunctival hyperemia in any of the study eyes. We observed superficial epithelium punctate keratitis in rabbits during the first hours with fluorescein sodium and lissamine green staining; however, it was not visible after 6 h of the first instillation of TALF. According to the *Pharmacopeia of Estados Unidos Mexicanos*, an ocular irritability test was satisfactory, and TALF is considered nonirritant. Additionally, in the phase I clinical assay, healthy volunteers reported predominantly minor and transient adverse events with TALF instillation, which supports the tolerability of the liposomal formulation.

Although further evaluation is needed to completely define the tolerability profile of this formulation, the preliminary results of our experimental study suggest that TALF is well tolerated and is safe in the eyes of New Zealand white rabbits, and it may have the potential to be used as a novel delivery system to reach the intraocular tissues of the posterior segment of the eyeball. These results can be considered useful for other topical liposome formulations comprising different steroids or other molecules. It is worth mentioning that the use of liposomal formulations through IVTs have demonstrated their effectiveness and safety in previous reports [59,60,61,62]. However, topical liposomes are a new approach for posterior ocular segment TA delivery when thinking about substituting IVT. Recently, Li, J and colleagues [15] have developed triamcinolone acetonide chitosan-coated liposomes as eye drops to deliver this steroid to the posterior segment of the eye. They have shown that this system has high entrapment efficiency (90.66%  ±  3.21%) and exhibits a sustained release profile, excellent physical stability, and no significant toxicity on the cornea, conjunctiva, and retina. Furthermore, Li, J and colleagues argued that their liposomal delivery platform is potentially more effective than our system since they showed higher concentrations of TA in posterior segment tissues. However, increased intraocular TA concentrations have shown major limitations, despite their increased effectiveness, concerning plausible unfavorable effects, including an elevation in and the formation of a posterior subcapsular opacity [4,5,6,63,64]. In our study we did not observe changes or opacities in the lenses of any eyes in the study groups and IOP also did not rise significantly in study eyes, but the therapeutic effect persisted in the retina (reduction of CFT). This background guided us to consider that TALF was safe and that it may have the potential to be a new drug delivery system for the posterior segment.

## 5. Conclusions

In conclusion, TALF has proven to be safe and well tolerated when used topically, and possesses therapeutic activity in humans. This represents a potential steroid therapy that could replace the use of IVTs of steroids, or could be used as an adjuvant to reduce the frequency of IVTs. In any case, it seems that complications related to intravitreal injections, as well as the adverse events related to the use of intraocular steroids, may be avoided by TALF. However, future larger experimental and clinical TALF studies to evaluate longer-term safety and the therapeutic profile are necessary.

## Figures and Tables

**Table 1:** Composition of triamcinolone acetonide-loaded liposome formulation (1 mL).

	(*w* or *v*)	(%)	
Triamcinolone acetonide	2.0 mg	0.2	*w*/*v*
Kolliphor HS 15	50 mg	5	*w*/*v*
PEG-12 glyceryl dimyristate	100 mg	10	*w*/*v*
Ethyl alcohol	14 mL	1.40	*v*/*v*
Citric acid anhydrous	0.8 mg	0.08	*w*/*v*
Sodium citrate dihydrate	4.675 mg	0.4675	*w*/*v*
Benzalkonium chloride	0.1 mg	0.01	*w*/*v*
Grade 2 purified water	Q.S.1.0 ml	NA	NA

NA; not applicable, *v*; volume, *w*, weight.

**Table 2:** Demographics and clinical characteristics of healthy subjects treated with triamcinolone acetonide-loaded liposomes formulation. BCVA, best corrected visual acuity; CFT, central foveal thickness; ETDRS, Early Treatment Diabetic Retinopathy Study; IOP, intraocular pressure; OD, right eye; OS, left eye.

				Baseline				14th Day			
Patient	Gender	Study Eye	Age	BCVA	Contrast	IOP	CFT	BCVA	Contrast	IOP	CFT
			(Years)	(ETDRS Letters)	(L/Contrast)	(mmHg)	(μm)	(ETDRS Letters)	(L/Contrast)	(mmHg)	(μm)
1	F	OD	25	85	1.65	10	250	85	1.65	12	248
2	F	OS	25	85	1.65	11	264	85	1.5	13	269
3	F	OD	26	85	1.35	10	256	85	1.35	16	260
4	F	OS	26	85	1.35	10	259	85	1.35	16	261
5	M	OD	25	83	1.35	13	242	84	1.5	14	243
6	M	OS	25	84	1.35	12	252	85	1.5	12	254
7	F	OD	24	84	1.35	11	243	85	1.5	8	247
8	F	OS	24	85	1.35	11	247	85	1.5	7	241
9	F	OD	24	85	1.5	16	255	85	1.5	11	258
10	F	OS	24	85	1.35	17	257	85	1.65	9	256
11	M	OD	56	85	1.5	16	257	85	1.65	15	256
12	M	OD	35	85	1.65	17	263	85	1.65	14	265
13	F	OD	47	85	1.65	13	246	85	1.65	15	245
14	F	OD	35	85	1.65	18	245	85	1.65	16	247
15	F	OD	63	83	1.35	12	258	85	1.65	14	260
16	M	OD	56	85	1.65	15	260	85	1.65	14	262
17	F	OD	40	85	1.65	16	262	84	1.65	14	259
18	F	OD	38	84	1.5	12	252	85	1.5	11	255
19	M	OD	63	84	1.5	16	249	84	1.5	13	248
20	M	OD	59	85	1.35	15	263	85	1.65	16	260
X ± s			37 ± 14.8	84.6 ± 0.6	1.5 ± 0.1	13.5 ± 2.7	254 ± 7	84.9 ± 0.3 ^‡^	1.6 ± 0.1 ^†^	13 ± 2.7 ^‡^	254.7 ± 7.8 ^‡^
*i_n_*	F = 13 M = 7	OD = 15 OS = 5									
*p*								0.096	0.014	0.4853	0.2825

in; frequency, F; female, M; male, OD; right eye, BCVA; best corrected visual acuity, CFT; central foveal thickness, ETDRS; Early Treatment Diabetic Retinopathy Study, IOP; intraocular pressure, TALF; triamcinolone acetonide-loaded liposomes formulation, ^†^; statistically significant differences from baseline values (*p* < 0.05), ^‡^; no statistically significant differences from baseline values (*p* > 0.05).

**Table 3:** Adverse events reported in healthy subjects treated with triamcinolone acetonide-loaded liposomes formulation.

Adverse Events	Categories	Dry Eyes	Burning	Discharge	Tearing	Blurred Vision	None
Safety Variables		n (%)	n (%)	n (%)	n (%)	n (%)	n (%)
Frequency	Not presented	14 (70)	14 (70)	18 (90)	17 (85)	18 (90)	8 (40)
	Rare	6 (30)	4 (20)				
	Occasionally		2 (10)		3 (15)		
	Most of the time			2 (10)		2 (10)	
	All the time						
Severity	Not presented	14 (70)	14 (70)	18 (90)	17 (85)	18 (90)	8 (40)
	Mild	6 (30)	6 (30)		3 (15)	2 (10)	
	Moderate			2 (10)			
	Severe						

TALF; triamcinolone acetonide-loaded liposomes formulation.

## Data Availability

The data presented in this study are available on request from the corresponding author. The data are not publicly available due to privacy and ethical restrictions.

## Abbreviations

AEs, adverse events; ARVO, Association for Research in Vision and Ophthalmology; BCVA, best corrected visual acuity; cECD, corneal endothelial cell density; CFT, central foveal thickness; CS, contrast sensitivity; DME, diabetic macular edema; ETDRS, Early Treatment Diabetic Retinopathy Study; F, female; FEUM, *Farmacopea de los Estados Unidos Mexicanos*; GMP, good manufacturing practice; HPLC, high-performance liquid chromatography; IBM, International Business Machines; IOP, intraocular pressure; IRB, Institutional Review Board; IVTs, intravitreal injections; LG, lissamine green; LPs, liposomes; M, male; MMPs, matrix metalloproteinases; OCT, optical coherence tomography; OD, right eye; OS, left eye; OSDI, ocular surface disease index; PEDF, pigment epithelium-derived growth factor; PRDL, Pharmaceutical Research and Development Laboratory; SAEs, serious adverse events; SEM, scanning electron microscopy; SPSS, Statistical Package for the Social Sciences; TA, triamcinolone acetonide; TALF, TA-loaded liposome formulation; TEM, transmission electron microscopy; VEGF, vascular endothelial growth factor.
